# Longitudinal panel networks of risk and protective factors for early adolescent suicidality in the ABCD sample

**DOI:** 10.1017/S0954579424001597

**Published:** 2024-10-10

**Authors:** Gemma T. Wallace, Bradley T. Conner

**Affiliations:** 1 Department of Psychiatry and Human Behavior, Warren Alpert Medical School of Brown University, Providence, RI, USA; 2 Department of Psychology, Colorado State University, Fort Collins, CO, USA

**Keywords:** early adolescent, family conflict, internalizing, network analysis, suicidal thoughts and behaviors

## Abstract

Rates of youth suicidal thoughts and behaviors (STBs) are rising, and younger age at onset increases vulnerability to negative outcomes. However, few studies have investigated STBs in early adolescence (ages 10–13), and accurate prediction of youth STBs remains poor. Network analyses that can examine pairwise associations between many theoretically relevant variables may identify complex pathways of risk for early adolescent STBs. The present study applied longitudinal network analysis to examine interrelations between STBs and several previously identified risk and protective factors. Data came from 9,854 youth in the Adolescent Brain Cognitive Development Study cohort (*M*_age_ = 9.90 ± .62 years, 63% white, 53% female at baseline). Youth and their caregivers completed an annual measurement battery between ages 9–10 through 11–12 years. Panel Graphical Vector Autoregressive models evaluated associations between STBs and several mental health symptoms, socioenvironmental factors, life stressors, and substance use. In the contemporaneous and between-subjects networks, direct associations were observed between STBs and internalizing symptoms, substance use, family conflict, lower parental monitoring, and lower school protective factors. Potential indirect pathways of risk for STBs were also observed. Age-specific interventions may benefit from prioritizing internalizing symptoms and early substance use, as well as promoting positive school and family support.

## Introduction

Suicidal thoughts and behaviors (STBs) are a critical public health problem among youth worldwide, and especially in the United States (National Institute of Mental Health, 2023). Suicide is the second-leading cause of death among individuals aged 10–14, and rates of death by suicide among adolescents (ages 15–19) alarmingly increased by 29% between 2014 and 2020 (United Health Foundation, 2023). STBs include suicidal ideation (i.e., thoughts about suicide) and suicidal behaviors (e.g., preparatory acts and interrupted, actual, and aborted suicide attempts) (Posner et al., 2011). Most youth STB research to date has been conducted in samples aged 13–18, but rates of STBs begin to increase after approximately age 10 (Nock et al., 2013). Although recent studies have investigated childhood STBs (DeVille et al., 2020; e.g., Harman et al., 2021; Janiri et al., 2020; Raffagnato et al., 2022), there is still a relative paucity of studies on STBs in early adolescence (ages 10–13) compared to older age groups (Ayer et al., 2020; de Sousa et al., 2017). Younger age at onset for STBs increases vulnerability to subsequent severe suicidality and mental health difficulties (e.g., Thompson et al., 2012). Thus, improving understanding of risk for STBs in early adolescence could inform early intervention efforts that could have protective impacts across the lifespan (Cha et al., 2018; Copeland et al., 2017; Reinherz et al., 2006).

In alignment with the Social-Ecological Framework, youth STBs are influenced by interrelations between risk and protective factors that occur at the individual (e.g., psychological constructs), interpersonal (e.g., family relationships), and environmental (e.g., neighborhood factors) levels (Cramer & Kapusta, 2017). Thus, it is important to consider risk and protective factors from multiple levels, including both individual differences and social-environmental factors, when assessing STB risk (see Carballo et al. (2020), Cha et al. (2018), and de Sousa et al. (2017) for reviews of STB risk and protective factors among youth). At the individual level, mental health symptoms are often identified as the most common risk factors for youth STBs. Depressive and other internalizing symptoms (e.g., negative cognitions) are salient risk factors (Carballo et al., 2020; Cha et al., 2018); however, STBs are a transdiagnostic phenomenon and have also been associated with sleep disturbances, externalizing, attention, thought, social, and other mental health symptoms (American Psychiatric Association, 2013; Carballo et al., 2020). Substance use is another common individual risk factor for youth STBs, which also occurs transdiagnostically across mental health symptoms (Carballo et al., 2020). At the interpersonal and environmental levels, several factors are robust correlates of youth STBs. In early adolescence, the family and school environment appear to have the largest impact on youth STBs, with lower family conflict, higher parental monitoring, and greater school engagement and support protecting against STBs (Carballo et al., 2020; Cha et al., 2018; Fotti et al., 2006; Janiri et al., 2020; Miller et al., 2015; Sedgwick et al., 2019; de Sousa et al., 2017). Peer and friendship factors appear to become more important as youth transition into adolescence (Telzer et al., 2018) and may be less salient during childhood and early adolescence. Additionally, youth STBs are often preceded by social-environmental stressors and adversity, such as negative life events (e.g., maltreatment, trauma, loss, and social stressors (e.g., bullying)), financial and material hardship, and perceptions of lower safety in a youth’s neighborhood (e.g., Cha et al., 2018; King et al., 2001; Pan & Spittal, 2013).

However, despite rigorous literature identifying these risk and protective factors, the etiological mechanisms underlying youth STBs remain unclear (Cha et al., 2018) and accurate prediction of future STBs is poor (Belsher et al., 2019; Franklin et al., 2017; Millner et al., 2020). This may be related to constraints of common study designs in psychiatric research that can obfuscate important information, including cross-sectional data (Cha et al., 2018; Guzmán et al., 2019), case-control frameworks (Caspi et al., 2020), and use of predictive models that cannot account for interrelations between many risk and protective factors (de Beurs, 2017). STBs appear to result not from one or a few predisposing factors, but from complex interplay between many risk and protective factors (Carballo et al., 2020; Cha et al., 2018; Fazel & Runeson, 2020; Millner et al., 2020; de Beurs, 2017). Thus, considering how risk and protective factors from multiple life domains interrelate with each other to influence STBs could increase understanding of how risk for STBs emerges and progresses across early adolescence (Cramer & Kapusta, 2017).

The network approach to psychopathology offers a useful framework for studying how interrelations between different risk and protective factors may influence STBs. The network approach posits that instead of resulting from *one shared cause*, mental health symptoms may have causal relations *with each other* (Borsboom, 2017; Fried et al., 2017). Thus, the network approach to psychopathology conceptualizes mental health concerns as emergent from interactive systems of psychological factors. The network approach is based on graph theory, in which psychological variables represent *nodes* and the pairwise associations between nodes (i.e., the strength of the association between each pair of variables) represent *edges* (Menczer et al., 2020). The network structure can be visualized graphically and statistically analyzed to identify patterns among the nodes (Epskamp et al., 2018; Hevey, 2018; Menczer et al., 2020). While network models cannot be interpreted as causal because they are based on observational data, they can be used to generate causal hypotheses about complex interrelations between several variables of interest (Borsboom & Cramer, 2013).

Approaching the study of STBs from a network perspective may increase understanding of how complex risk and protective pathways emerge (de Beurs, 2017). By estimating bivariate associations between each pair of variables while adjusting for all other variables in the model, network models provide valuable information about the structure of relationships between variables in high-dimensional data. This multivariate structure is not as clearly revealed in other analytic approaches; while multiple regression models provide similar statistical information regarding the prediction of specific outcomes, network models have the advantage of providing clear and powerful visualizations of the multivariate structure for all variables in the model (Borsboom et al., 2021). This can identify nodes that are highly or sparsely associated with the other nodes in the network (Epskamp et al., 2018; Menczer et al., 2020). For example, certain nodes may have strong connections with many other nodes, suggesting they may have the ability to activate or suppress the network as a whole. Other nodes may have only one or a few connections with other nodes and may peripherally influence (or be influenced by) the network (Borgatti, 2005). Thus, network models can provide information about complex patterns of conditional dependence in multivariate data. The network approach may therefore help to map both direct and potential indirect pathways of risk among previously identified risk and protective factors for STBs (Shiratori et al., 2014; de Beurs, 2017).

A small but growing body of literature has applied network approaches to the study of youth STBs (Fonseca-Pedrero et al., 2020, 2022; Gijzen et al., 2021; Li & Kwok, 2023; Ou et al., 2023; Zhong et al., 2023). Most studies to date have focused on STBs’ relations with psychological constructs only, such as mental health symptoms and cognitive-affective constructs (e.g., Fonseca-Pedrero et al., 2020; Gijzen et al., 2021; Li & Kwok, 2023). For example, cross-sectional associations were observed between suicidal ideation, depressive symptoms, affect, and self-esteem in a sample aged 13–16 years (Fonseca-Pedrero et al., 2020). In another study, subjective happiness and hopelessness, moderated by self-efficacy, were prospectively associated with STBs across two timepoints among adolescents aged 9–15 years (Li & Kwok, 2023). Less research has extended network models to consider domains of STB risk and protective factors other than psychological constructs (e.g., socioenvironmental factors and stressors). Cross-sectional network studies have identified direct and potential indirect associations between STBs, mental health symptoms, and problems with peers and/or bullying in samples of adolescents aged 12–20 (Zhong et al., 2023) and 14–80 (Fonseca-Pedrero et al., 2022). Another recent study estimated cross-sectional networks of adolescent psychological constructs and parenting styles (Ou et al., 2023). These important studies substantiate that STBs involve complex systems of risk and protective factors. However, the cross-sectional or two-timepoint designs of previous research preclude evaluating longitudinal and within-person associations between STBs and risk and protective factors. To our knowledge, no previous research has employed longitudinal network analyses to investigate how risk and protective factors from multiple life domains relate to STBs across early adolescence.

### Present study

The goal of this study was to examine how risk and protective factors from multiple life domains relate to each other, both within and across domains, and to STBs during the transitional period from childhood into early adolescence. We applied a longitudinal network perspective to examine pairwise relations between several previously identified correlates of STBs in a cohort of 9,854 youth followed from ages 9–10 to 11–12 years as part of the Adolescent Brain Cognitive Development (ABCD) Study. We evaluated several constructs that have been robustly identified as STB risk and protective factors, including mental health symptoms (internalizing, externalizing, attention problems, social problems, thought problems, and sleep problems), socioenvironmental factors (family conflict, parental monitoring, and school protective factors), stressors (stressful life events, material hardship, and neighborhood safety), and substance use (e.g., Carballo et al., 2020; Cha et al., 2018; de Sousa et al., 2017). Most of these constructs have also been identified as STB risk and protective factors in cross-sectional studies of the ABCD sample specifically, increasing confidence in their salience (DeVille et al., 2020; Harman et al., 2021; Janiri et al., 2020; van Velzen et al., 2021). To our knowledge, this study represents the first application of a longitudinal panel network approach to examine early adolescent suicidality. Illuminating pathways of direct and potential indirect risk for early onset STBs could identify early predisposing factors and inform the timing and targets of specific interventions. Given that network models are data driven, we did not make causal hypotheses about effect directions and expected that the models would identify new and complex relations among the risk and protective factors for STBs.

## Methods

### Participants and procedures

The ABCD study design and protocols have been described in detail elsewhere (Auchter et al., 2018; Feldstein Ewing et al., 2018; Garavan et al., 2018; Karcher & Barch, 2021; Saragosa-Harris et al., 2022). Currently in its fifth year of data collection, the same cohort will be followed longitudinally for 10 years, from ages 9–10 to 19–20 years. A comprehensive measurement battery is administered annually, with parent- and youth-report measures of mental and physical health, culture and environment, neurocognition, and substance use. Study protocols are provided at https://abcdstudy.org/scientists/protocols/. The present study used data from the first three annual waves: Baseline (aged 9–10 years, *N* = 11,878), Year 1 (aged 10–11 years, *N* = 11,235), and Year 2 (aged 11–12 years, *N* = 10,414).

The ABCD study oversampled siblings (Garavan et al., 2018; Karcher & Barch, 2021), and the baseline sample included 8,150 singletons, 1,600 non-twin siblings, 2,100 twins, and 30 triplets (Palmer et al., 2021). To avoid nested data within households, one sibling was randomly selected from each family (i.e., the sample consisted of unrelated participants). This resulted in an analytic sample of *N* = 9,854 at baseline, *N* = 9,286 at Year 1, and *N* = 8,629 at Year 2. Table 1 presents demographic characteristics and rates of STBs in the analytic sample, as well as the full ABCD sample before dropping sibling participants. Sample characteristics were highly consistent across the original and analytic samples. At each timepoint, 8.0–8.7% of youth reported any form of STBs.

### Measures

The ABCD measurement battery has been described in detail (see Barch et al. (2017) for details on the mental and physical health measures, Lisdahl et al. (2018) for substance use measures, Zucker et al. (2018) for socioenvironmental measures, and Hoffman et al. (2019) for stress exposure measures). Our analyses included constructs available in the ABCD data that (1) previous literature identified as a theoretically relevant risk or protective factor for youth STBs, and (2) had available data from all three annual waves and from one consistent reporter, either parent or youth (see Table 2 for the list of variables included in the present study).

### Suicidal thoughts and behaviors

Youth report of STBs was measured with the Suicide Module of the computerized Kiddie Schedule for Affective Disorders and Schizophrenia for the DSM-5 (KSADS-COMP) (KSAD-COMP LLC, 2024 Townsend et al., 2020). Participants responded to binary items assessing the presence (1) or absence (0) of nine STBs: passive suicidal ideation, active but non-specific suicidal ideation, suicidal ideation with a specific method, active suicidal ideation with intent, active suicidal ideation with a plan, preparatory actions toward suicidal behavior, interrupted suicide attempt(s), aborted suicidal attempt(s), and suicide attempt(s). While different forms of STBs have different implications for intervention (e.g., passive ideation versus attempt) (Klonsky et al., 2017; Nock et al., 2013), any level of STBs in this age range is clinically concerning and confers vulnerability to long-term negative outcomes (Cha et al., 2018; Thompson et al., 2012). Thus, suicidality was modeled as one variable reflecting a count of the number of STB items a youth endorsed at each wave (i.e., ever at baseline, or since the previous measurement occasion at Years 1–2). Previous research using the ABCD sample used a similar coding scheme, in which suicidality was defined as endorsement of one or more KSADS STB items (Janiri et al., 2020).

### Mental health symptoms

Parent/caregiver report of their child’s mental health symptoms over the past 6 months was measured with the ASEBA Child Behavior Checklist (CBCL) (Achenbach, 2009). Parents responded to each item on a 3-point scale of 0 = *not true (as far as you know)*, 1 = *somewhat or sometimes true*, and 2 = *very true or often true*. Raw scores were calculated for the following CBCL Syndrome Scales (Achenbach, 2009): Social Problems (11 items, e.g., “Complains of loneliness”; within *ω* = .53, between *ω* = .85), Thought Problems (11 items, e.g., “Can’t get their mind off certain thoughts”; within *ω* = .44, between *ω* = .82), and Attention Problems (10 items, e.g., “Fails to finish things they start”; within *ω* = .69, between *ω* = .94). Internalizing was a sum of 12 items from the Anxious/Depressive syndrome scale (e.g., “Fears going to school”), eight items from the Withdrawn/Depressed scale (e.g., “Would rather be alone than with others”), and 10 items from the Somatic Complaints scale (e.g., “Feels dizzy or lightheaded”) (30 items total; within *ω* = .80, between *ω* = .93). Externalizing was a sum of 14 items from the Rule Breaking scale (e.g., “Doesn’t seem to feel guilty after misbehaving”) and 18 items from the Aggressive Behavior scale (e.g., “Cruelty, bullying, or meanness to others”) (32 items total; within *ω* = .85, between *ω* = .96) (Achenbach, 2009). To avoid latent confounding (Hallquist et al., 2021), CBCL items that directly overlapped with other study variables were omitted, including the two STB items, three substance use items, and two sleep problem items. Coding schemes for the CBCL variables are provided in the Appendix.

Parent/caregiver reports of their child’s sleep problems over the past 6 months were measured using the Sleep Disturbances Scale for Children (SDSC) (Bruni et al., 1996), a 26-item scale assessing six domains of sleep-related problems (e.g., “The child has difficulty getting to sleep at night”). Parents responded to items on a 5-point scale of 1 = *never* to 5 = *always* (*daily*), where higher values reflected more sleep difficulties. Sleep problems were modeled as a total sum score of the 26 SDSC items (within *ω* = .82, between *ω* = .85).

### Socioenvironmental factors

Youth report of perceived family conflict was assessed with the PhenX Family Conflict subscale of the Family Environment Subscale (Moos & Moos, 1994). Youth responded to nine items on a binary scale where 0 = *false* and 1 = *true* (e.g., “We fight a lot in our family”). Family conflict was modeled as a sum score of the nine items (within *ω* = .51, between *ω* = .90), with higher scores representing higher levels of family conflict.

Youth report of perceived parental monitoring was measured with the ABCD Parental Monitoring Survey (Karoly et al., 2016; Stattin & Kerr, 2003). Youth responded to five items on a 5-point Likert-style scale of 1 = *never* to 5 = *always or almost always* (e.g., “How often do your parents/guardians know where you are?”). Parental monitoring was modeled as a sum score of the five items, and items were reverse coded such that higher values reflected lower parental monitoring (within *ω* = .35, between *ω* = .75).

Youth report of school protective factors was measured using the PhenX School Risk and Protective Factors Survey (SRPF) (Arthur et al., 2007). The SRPF includes three subscales that measure youth’s perception of their school environment (6 items, e.g., “In my school, students have lots of chances to help decide things like class activities and rules”), school involvement (4 items, e.g., “In general, I like school a lot”), and school disengagement (2 items, e.g., “Usually, school bores me”). Youth responded on a 4-point scale of *1* = *NO!, 2* = *no, 3* = *yes, and 4* = *YES!* School protective factors was a sum score of the 12 SRPF items, where higher values represented higher levels of school protective factors (school disengagement items were reverse coded; within *ω* = .77, between *ω* = .89).

### Stressors

Parent/caregiver report of neighborhood safety was assessed using the PhenX Neighborhood Safety/Crime Survey (Echeverria et al., 2004; Mujahid et al., 2007). Parents responded to three items on a 5-point Likert-style scale of 1 = *strongly disagree* and 5 = *strongly agree* (e.g., “I feel safe walking in my neighborhood, day or night”). Neighborhood safety was a sum score of the three items (within *ω* = .77, between *ω* = .95); items were reverse coded such that higher scores represented lower neighborhood safety.

Parent/caregiver report of family material hardship was measured in the PhenX Demographics Survey (Barch et al., 2017). Parents responded to seven binary items about types of material hardship their family had experienced in the past 12 months due to financial difficulties (0 = *no*, 1 = *yes*; e.g., “In the past 12 months, has there been a time when you and your immediate family needed food but couldn’t afford to buy it or couldn’t afford to go out to get it?”). Material hardship was a count of the number of material hardship items parents/caregivers endorsed, where higher values reflected more types of material hardship.

Parent/caregiver report of stressful life events their child experienced was measured as a composite of items from the KSADS-COMP Post Traumatic Stress Disorder (PTSD) module (KSAD-COMP LLC, 2024 Townsend et al., 2020) and the Life Events Scale (LES) (Grant et al., 2004; Hoffman et al., 2019). The KSADS PTSD module asked parents if their child had experienced 17 types of potentially traumatic events (0 = *no*, 1 = *yes*; e.g., “Learned about the sudden unexpected death of a loved one”). The KSADS PTSD module was scored as the number of events parents endorsed their child having ever experienced at baseline, or since the previous measurement occasion at Year 2. The LES similarly assessed parents’ knowledge about 26 significant life experiences their child experienced in the past year, and if the event was “mostly good” or “mostly bad” (e.g., “Someone in family died”). The LES was scored as the number of “mostly bad” events parents endorsed for their child. The KSADS PTSD module was administered to parents at baseline and Year 2 (i.e., not available for Year 1). The LES was administered to parents at Years 1–2 only (i.e., not available at baseline). Given that stressful life events are salient risk factors for STBs, these measures were integrated to provide a proxy for youth’s stressful life events at all three waves. Stressful life events were modeled as the KSADS PTSD score at baseline, the LES score at Year 1, and the mean of the KSADS PTSD and LES scores at Year 2. The raw KSADS PTSD and LES scores were positively correlated (*ρ* = 0.37), and each score was standardized to account for different response scales on the two measures. While the KSADS PTSD module and LES have content overlap for several items, they do not assess identical stressors. Results that involve this variable should be interpreted with these considerations in mind.

### Substance use

Youth report of substance use was measured by the ABCD Timeline Follow-back Interview (TLFB) (Lisdahl et al., 2018). The young age of participants (10–12 years) is before age at onset for most substance use, and rates of engagement in moderate substance use were low in the ABCD cohort (Lisdahl et al., 2018; Martz et al., 2022). Thus, we focused on low-level use (e.g., a sip or puff) of the three most used substances: alcohol, nicotine, and cannabis (Lisdahl et al., 2021; Martz et al., 2022). Youth were first asked if they had ever heard of several substances. If they responded yes, they were then asked if they had tried each substance (0 = *no*, 1 = *yes*). Alcohol use was assessed with “Have you ever tried a sip of alcohol such as beer, wine or liquor (rum, vodka, gin, whiskey)?” Nicotine use was assessed with “Have you ever tried a puff from a tobacco or electronic cigarette, Juul, vape pens, e-hookah, cigar or pipe?” Cannabis use was assessed with “Have you ever tried a puff or eaten any marijuana, also called pot, grass, weed or ganja?” Substance use was modeled as a count of the three substances youth reported having tried (measured as ever having used at baseline, or having used since the last measurement occasion at Years 1–2).

### Statistical analyses

Data wrangling and analyses were conducted in R version 4.3.0 (R Core Team, 2023). Within-person omegas are typically lower than between-person omegas in multilevel data, especially when the number of measurement occasions is small (three in the present study) (Geldhof et al., 2014; Rush & Hofer, 2017). All variables were assessed for multivariate normality and stationarity assumptions, and multilevel omegas were calculated for scale-scored variables (Wiley, 2020). Prior to analysis, all variables were standardized (i.e., *z*-scored) across all three waves of data (while in a long format). Because youth age is a theoretically relevant covariate of both STBs and the risk and protective factors (e.g., Smetana & Rote, 2019), data were detrended for the linear effects of age. A series of bivariate linear regressions were conducted in which each variable was regressed on age, and the resulting residuals for each variable were modeled as nodes in the networks (Burger et al., 2022; Fried et al., 2022; Ram et al., 2013). Regression coefficients for age predicting each variable are shown in Supplemental Table S1. Sensitivity analyses using non-detrended versions of study variables indicated that detrending did not meaningfully impact the results (O’Driscoll et al., 2022).

Network analyses were conducted using Panel Graphical Vector Autoregressive (Panel GVAR) models via the *psychonetrics* package (Epskamp, 2020, 2022), which enables estimation of temporal network models in panel data with three or more timepoints. We encourage readers to review Epskamp (2020) for detailed descriptions of Panel GVAR models and example R code for the *psychonetrics* package. Panel GVAR models integrate multivariate modeling with Gaussian Graphical Models, a type of network model for continuous data, and Graphical Vector Autoregressive Models, a type of model for estimating multilevel temporal effects (Epskamp, 2020). In our Panel GVAR model, the network *nodes* (i.e., variables) were suicidality and the 13 different risk and protective factors (14 nodes total, listed in Table 2). All nodes were modeled as observed (i.e., non-latent) variables. Network *edges* represented the statistical associations between each pair of nodes after adjusting for all other variables in the model. In our Panel GVAR model, all edges were estimated as fixed (i.e., not allowed to randomly vary across participants) partial correlations between each pair of nodes (Epskamp & Fried, 2018). *Undirected edges* represented bidirectional partial correlations between variables within the same timepoint, while *directed edges* represented predictive (unidirectional) partial correlations between variables across timepoints (Hevey, 2018).

Panel GVAR models simultaneously estimate three network structures: temporal, contemporaneous, and between-subjects networks (Epskamp, 2020; Jordan et al., 2020). The temporal network provides directed within-person lagged partial correlations and autocorrelations across timepoints. Edges in the temporal network are considered predictive because they represent a variable from a previous timepoint predicting either itself (autocorrelation) or another variable (bivariate correlation) at a subsequent timepoint. The contemporaneous network provides undirected within-person partial correlations between each pair of nodes within the same timepoint, after controlling for temporal effects. In other words, these are bidirectional associations rather than predictive relations. Lastly, the between-subjects network provides undirected partial correlations that reflect associations between overall variable means across all timepoints. Again, these are bidirectional associations rather than predictive relations (see Epskamp (2020) for detailed descriptions of the three network structures). Panel GVAR models used the nlminb optimizer (Epskamp, 2022), and missing data was handled using Full Information Maximum Likelihood (FIML). Network structures were visualized using *qgraph* (Epskamp et al., 2012, 2023).

### Model selection and evaluation

Model fit was evaluated using multiple indices: root mean square error of approximation (RMSEA), comparative fit index (CFI), Tucker-Lewis index (TLI), and Bayesian information criterion (BIC). Model *χ*^2^ was also reported but not interpreted due to sample size sensitivity (Tabachnick & Fidell, 2013). Panel GVAR models are relatively new, and there are not well-established criteria for evaluating model fit, but recommendations for structural equation models can be used as guidelines (Epskamp, 2020; O’Driscoll et al., 2022). To select the best-performing model, edge selection was conducted via recursive step-down and step-up model searches. In the step-down pruning step, edges not significant at *p* < .01. were iteratively removed and the model was refit with these edges fixed at 0 (Blanken et al., 2022). In the subsequent step-up search, edges were iteratively added until the best-performing BIC was obtained (Blanken et al., 2022; Epskamp, 2020). To assess stability of the edges, we used a 25% case-drop bootstrap resampling procedure (Epskamp, 2020), in which 100 models were refit using a random 75% of the sample. Edges retained in higher proportions of bootstrapped models are considered more stable (Epskamp et al., 2018).

Node importance was evaluated via centrality metrics, including node strength, betweenness, and closeness (Epskamp et al., 2018; Epskamp & Fried, 2018; Hevey, 2018). Node *strength*, calculated as the sum of all absolute edge weights connected to a node, represents how strongly a node directly relates to other nodes in the network. High strength suggests a node has strong relations with other variables in the network. Closeness and betweenness quantify a node’s indirect associations with all other nodes in the network. *Closeness* represents the average shortest path (measured by geodesic distance [Opsahl et al., 2010]) between a specific node and all other nodes. *Betweenness* represents the number of times a node is on the shortest path (measured by geodesic distance) between other nodes. High closeness and betweenness suggest nodes can indirectly influence and be influenced by other nodes in the model (Deserno et al., 2022; Hevey, 2018). All centrality indices were *z*-scored, and stability of the centrality indices was assessed in the case-drop bootstrap resampling procedure.

## Results

Descriptive statistics and rates of missingness for study variables are presented in Table 3. Multilevel bivariate correlations and intraclass correlations for study variables are shown in Table 4. Variables were generally non-normal and had missing data for 6.41–8.60% of cases (Table 3). Although FIML assumes multivariate normality, simulation studies suggest it can be robust to non-normality, particularly in large sample sizes (Jobst et al., 2021). The bootstrapping procedure can be used to evaluate the stability of network analysis results when variables are non-normal (Epskamp, 2020).

### Model results

**Figure 1.**
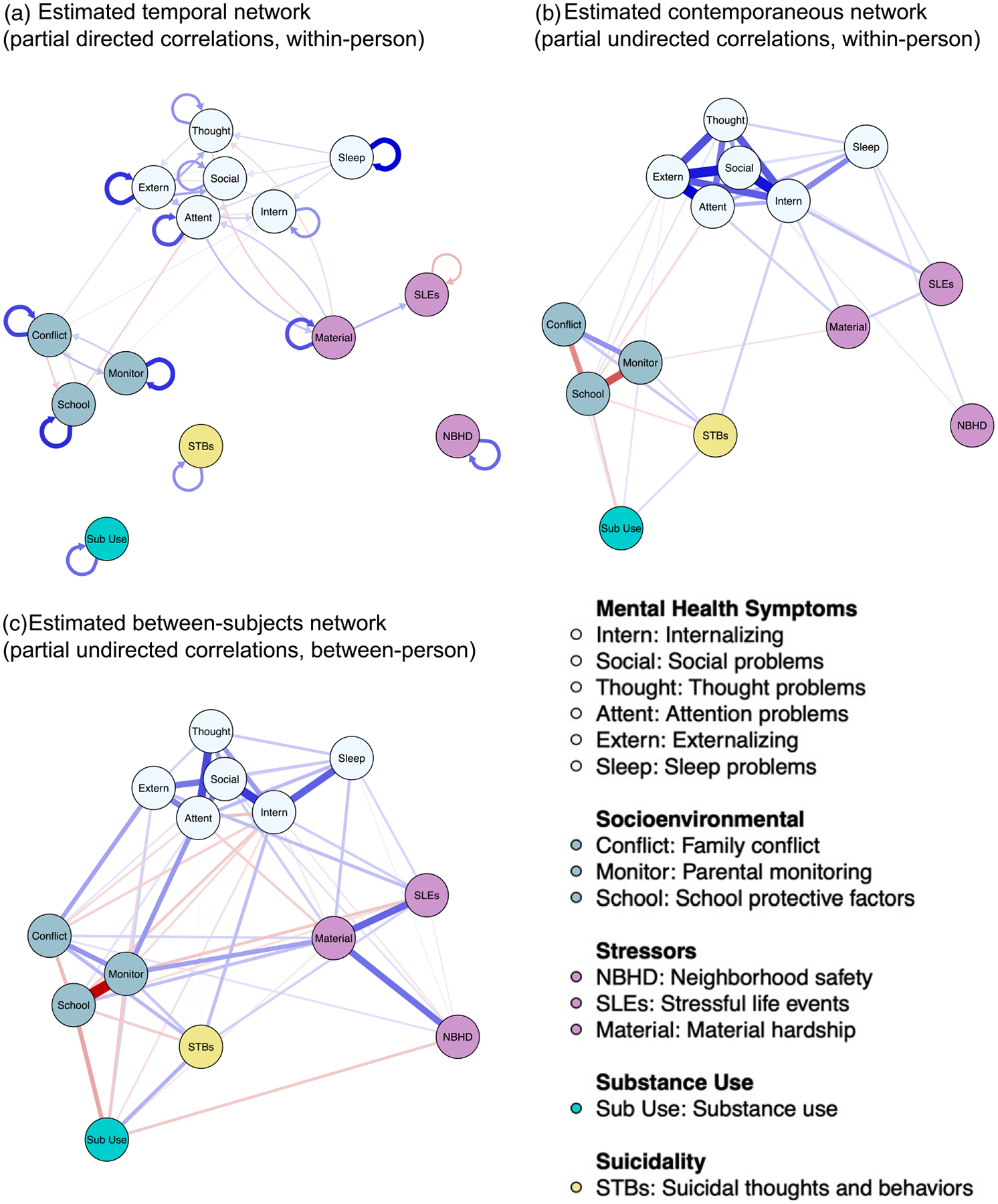
Pruned network structures for the panel GVAR model (*N* = 9,854). Edge color represents effect direction (blue = positive, red = negative), while edge thickness represents effect strength (darker, thicker edges denote larger effects). Edges not shown were pruned during model selection. (a) Arrows represent lagged directed partial correlations and autocorrelations in the temporal network. (b) Lines represent undirected partial correlations in the contemporaneous network. (c) Lines represent undirected partial correlations in the between-subjects network. Corresponding numeric results are presented in Tables 5–6.

**Figure 2.**
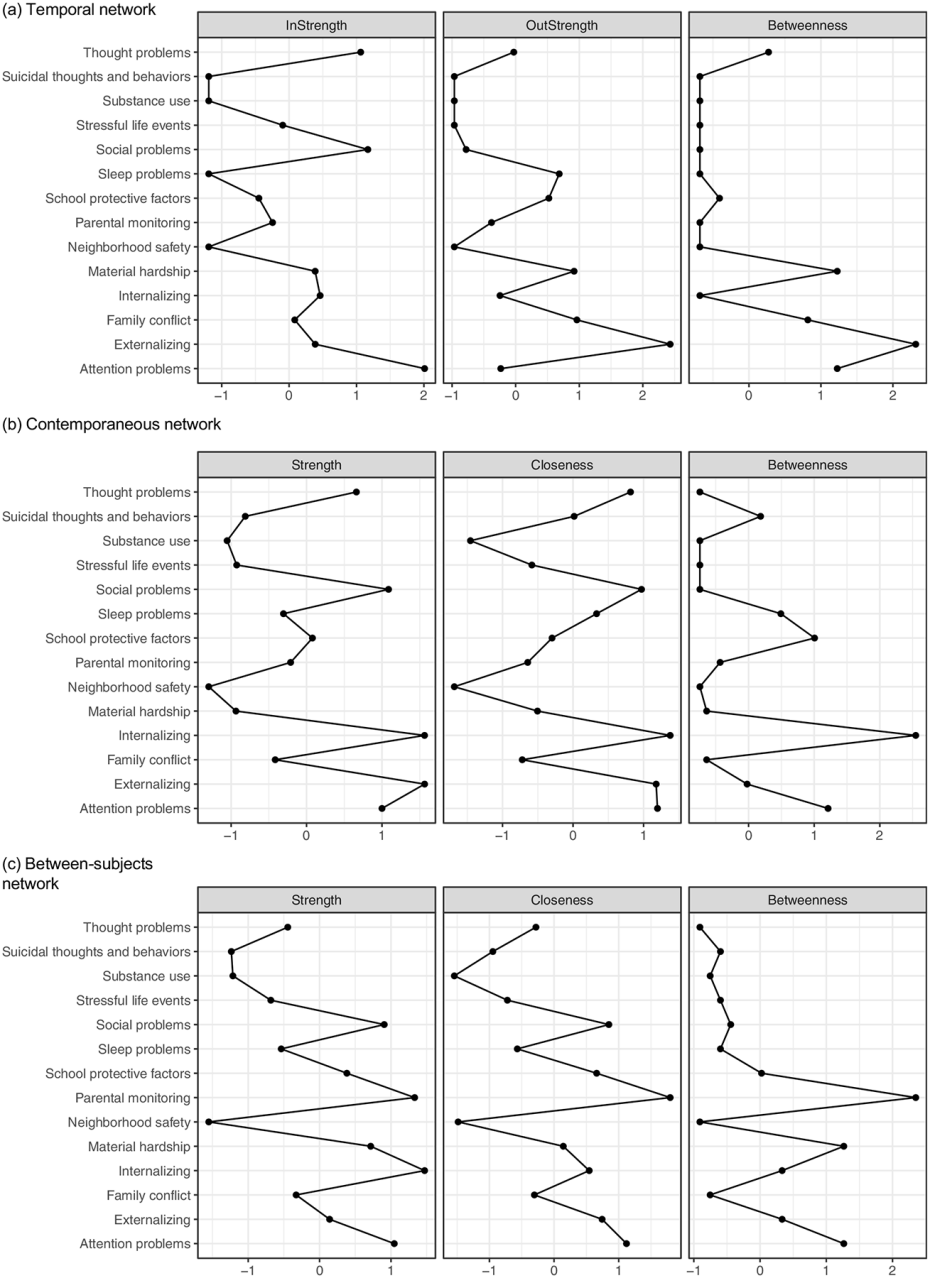
Node centrality metrics for the panel GVAR model in the full sample (*N* = 9,854). Centrality metrics are shown in the metric of *z*-scores. (a) In the temporal network, In-Strength centrality represents the sum of all incoming absolute edge weights to a node, while Out-Strength centrality represents the sum of outgoing absolute edge weights from a node. (b–c) In the contemporaneous and between-subjects networks, Strength centrality represents the sum of all absolute edge weights connected to a node. Closeness represents the average shortest path between a specific node and all other nodes. Betweenness represents the number of times a node is on the shortest path between other nodes (Hevey, 2018).

The model demonstrated excellent fit to the data: BIC = 903970.85, RMSEA = 0.03, CFI = 0.97, TLI = 0.96, *χ*^2^ [df = 764] = 7304.65 (*p* < 0.05). Network structures are shown in Figure 1. Corresponding correlation coefficients for the temporal, contemporaneous, and between-subjects networks are presented in Tables 5–6, respectively. The bootstrap inclusion probabilities for each edge are shown in Supplemental Table S2. Centrality indices, including strength, betweenness, and closeness, for the full sample model are shown in Figure 2. Supplemental Figure 1 presents average centrality values for the network structures across the 100 case-dropped random subsamples from the bootstrapped models. Given the emphasis of the present study, we focused our interpretation on direct and potential indirect associations between STBs and other nodes in the networks.

In the pruned, estimated temporal network, autocorrelations were observed for all nodes in the network (Figure 1a; Table 5). The bootstrapping procedure indicated high stability of all autoregressive effects (observed in ≥ 92% of bootstrapped models; Supplemental Table S1). Thus, higher STBs at an earlier timepoint were associated with higher STBs at a later timepoint. Several lagged correlations were also observed between the mental health symptom, socioenvironmental, and stressor nodes. STBs were not directly associated with other nodes in the temporal network. With regard to centrality, attention problems, social problems, and thought problems had the highest in-strength centrality, indicating relatively strong predictive effects on other nodes in the model (Figure 2a). Externalizing, family conflict, and material hardship had the highest values for both out-strength and betweenness centrality, indicating they were most predicted by other nodes and had relatively strong indirect associations with other nodes in the network. However, centrality indices for the temporal network differed somewhat between the full sample and 25% case-drop bootstrapped models (Supplemental Figure 1), suggesting that node centrality for the temporal effects varied across different subsets of participants in the sample.

In the pruned, estimated contemporaneous network, direct associations were observed between STBs and five other nodes: internalizing symptoms, substance use, family conflict, lower parental monitoring, and lower school protective factors (Figure 1b; Table 6). The bootstrapping procedure indicated high stability of these direct effects (included in ≥ 89% of bootstrapped models; Supplemental Table S1). Potential indirect pathways to STBs were also observed. Internalizing was associated with the other five mental health symptom dimensions (externalizing, thought problems, attention problems, sleep problems, and social problems), material hardship, and stressful life events. Each of these internalizing edges was included in 100% of the bootstrapped models, suggesting internalizing may potentially be a pathway through which other mental health symptoms, material hardship, and stressful life events associate with suicidality. Further, pairwise edges were observed between family conflict, parental monitoring, school protective factors, and substance use, indicating elevation in one of these constructs associates with elevation in the others, which may increase overall risk for STBs. Mental health symptom nodes had the highest strength and closeness centrality, including internalizing, externalizing, attention problems, and social problems (Figure 2b). Internalizing, attention problems, and school protective factors displayed the highest betweenness centrality. The centrality indices for the contemporaneous network were largely consistent between the original model and the bootstrap resampling procedure (Supplemental Figure 1), indicating stability of these indices.

The overall structure of the between-subjects network was similar to the contemporaneous network, although additional edges were observed (Figure 1c; Table 6). STBs again had direct associations with internalizing symptoms, substance use, family conflict, lower parental monitoring, and lower school protective factors. An additional direct association was observed between STBs and stressful life events. The bootstrapping procedure generally indicated effect stability, although the edge between STBs and parental monitoring was only observed in 31% of the bootstrapped models (Supplemental Table S1). Internalizing, parental monitoring, and attention problems had the highest strength centrality (Figure 2c). Parental monitoring and attention problems displayed the highest closeness centrality, while parental monitoring, material hardship, and attention problems had the highest betweenness centrality. Centrality indices for the between-subjects network were highly consistent across the original model and the bootstrap resampling procedure (Supplemental Figure 1).

## Discussion

The present study applied a longitudinal network approach to elucidate potential pathways of risk and protection for STBs during the critical developmental period from late childhood into early adolescence, an age range that has been relatively understudied in youth STB research. Models examined risk and protective factors for STBs from multiple salient life domains, including mental health symptoms, socioenvironmental factors, life stressors, and substance use. Risk and protective factors representing multiple life domains directly associated with STBs, highlighting the value of applying a social-ecological lens to youth STB research (Cramer & Kapusta, 2017). Further, potential indirect pathways to STBs were observed, in which constructs that are directly associated with STBs are also associated with other risk and protective factors in the network. By elucidating the multivariate structure of STBs and several risk and protective factors, our results identified potential pathways through which interrelations between different risk and protective factors may influence early adolescent STBs.

### Effect timescales and sizes

It is important to consider timescales when interpreting longitudinal effects (Burger et al., 2022; Epskamp, 2020). In the present study, measurement occasions were spaced approximately 1 year apart. However, the timescales for the causal effects of many risk and protective factors on STBs may be shorter than 1 year (Berman & Silverman, 2014). For example, suicidality commonly co-occurs with internalizing symptoms (Goldston et al., 2009). Past-year STBs would hence be expected to have a stronger association with internalizing symptoms assessed during the same timepoint (in this case, the CBCL measured past 6-month symptoms), compared to internalizing symptoms measured 1–2 years previously. The contemporaneous network structure is therefore most likely to capture the putatively causal associations between variables in our model (Burger et al., 2022). The lack of robust associations between STBs and other variables in the temporal networks indicates that the 1-year time-lag between measurement occasions may be too long to capture meaningful impacts of the other variables on STBs. Moreover, the presence of several consistent contemporaneous effects with mostly null temporal effects suggests that the effects of other variables on STBs may diminish over time. Thus, while it is important to control for temporal lagged and autoregressive effects in the networks (Usami et al., 2019), we primarily focus our interpretation on the contemporaneous network structure. Further, observed effect sizes were generally small (*r* < .3) in both full and partial correlations between study variables. This is consistent with other publications using the ABCD data, in which effect sizes are smaller than what would be expected given previous literature on the measured variables (Gonzalez et al., 2021; Owens et al., 2021).

### Direct pathways of risk and protection for STBs

In contemporaneous network structure, STBs had direct, positive associations with five risk and protective factors: higher family conflict, lower parental monitoring, lower school protective factors, higher internalizing symptoms, and higher substance use. While these risk and protective factors have been robustly identified in previous STB literature, observing these effects in network models bolsters confidence in their importance; these effects were consistent after controlling for all other pairwise associations in the networks. Thus, results emphasize that family and school environments serve as contexts for salient social experiences in this age range (Carballo et al., 2020; Cha et al., 2018; Fotti et al., 2006; Janiri et al., 2020; Miller et al., 2015; Sedgwick et al., 2019; de Sousa et al., 2017). Youth who perceived higher levels of expressed emotion and aggression in their family, and their caregivers being less involved in and aware of their daily activities, were more likely to report STBs. Additionally, youth who reported lower engagement and support in their schools were at greater risk for STBs.

It is interesting that internalizing symptoms were directly associated with STBs while other mental health symptom dimensions were not. Previous literature has emphasized the transdiagnostic and comorbid nature of STBs (American Psychiatric Association, 2013; Carballo et al., 2020; Cha et al., 2018), and other mental health symptom dimensions (e.g., externalizing) have been associated with STBs in studies of the ABCD cohort (DeVille et al., 2020; Harman et al., 2021; Janiri et al., 2020; van Velzen et al., 2021). However, these studies did not use a network approach that adjusted for temporal and other pairwise effects in models. Among early adolescents, internalizing may be a more important mental health risk factor for STBs compared to other symptom dimensions. This finding warrants replication in other samples.

The direct association between substance use and STBs is also noteworthy. Both STBs and substance use can be harmful in this age range, and the co-occurrence of STBs and substance use during early adolescence may compound the risk for negative long-term outcomes (Effinger & Stewart, 2012). STBs also had a direct effect with stressful life events in the between-subjects network, suggesting that, overall, youth with a lifetime history of stressful life events were more likely to report lifetime history of STBs. This is in line with previous research identifying adverse life events as correlates of STBs (Cha et al., 2018; King et al., 2001; Pan & Spittal, 2013). Lastly, the observed autoregressive effect for STBs in the temporal network affirms that individuals with a history of STBs have elevated risk for future STBs, and risk assessment over time should account for this (Robinson et al., 2018).

### Potential indirect pathways of risk and protection for STBs

Potential indirect pathways to STBs were also observed in the contemporaneous network structure. While internalizing was the only mental health node that exhibited a direct association with STBs, internalizing had high centrality and was associated with the other five mental health symptom dimensions, stressful life events, and material hardship. Thus, internalizing may *potentially* provide a pathway through which life stressors and other mental health symptoms contribute to risk of STBs. For example, externalizing problems could potentially lead to criticism from others, which could negatively impact mood and self-esteem, thereby contributing to thoughts of suicide. It might be the case that having externalizing symptoms only increases the propensity for experiencing STBs if individuals have co-occurring internalizing symptoms, thus explaining some of the inconsistencies in the literature examining links between externalizing symptoms and STBs (Piqueras et al., 2019; Witte et al., 2018). Further, stressful life events may be more likely to impact STBs if youth experience resulting internalizing difficulties. However, because these effects were in the contemporaneous network, we cannot make assumptions about the temporal order of associations between internalizing, these other risk factors, and STBs.

Pairwise associations were also observed between substance use and all three of the socioenvironmental risk factors (family conflict, parental monitoring, and school protective factors). Because these factors are all related to STBs and to each other, results suggest the potential for a feedback system in which these factors could conjointly increase risk for STBs. For example, low parental monitoring can increase a youth’s risk for early initiation of substance use (Dever et al., 2012) and poorer school performance and engagement (Lowe & Dotterer, 2013). This could potentially contribute to higher family conflict, which could reinforce ongoing school and substance use concerns (Timmons & Margolin, 2015). Thus, activation of one or more of these risk factors may increase the other risk factors, exacerbating and potentially perpetuating overall risk for STBs (Borsboom, 2017; Dablander et al., 2020). However, because these variables were all related, intervening on one of these risk factors might have protective effects on the other nodes. Hence, in addition to direct effects, interrelations *between* risk and protective factors may impact the development and course of STBs. Many of these effects may be obscured in traditional models that do not consider pairwise relations between all variables. Thus, the present study supports the use of longitudinal network analyses to provide information about complex systems of risk and protection for STBs (de Beurs, 2017). Nonetheless, our use of observational data precludes interpreting these potential indirect pathways as causal. We encourage future research to prospectively test these indirect effects in mediation models and to use shorter measurement timescales that are more likely to capture the causal timeframe of these effects (i.e., less than 12 months apart).

### Clinical implications

To date, relatively few suicide-focused interventions have been specifically tailored to late childhood and early adolescence (Robinson et al., 2018). Our results identified risk and protective factors that are directly related to STBs and could be amenable to prevention and early intervention initiatives (McGorry & Mei, 2018). Internalizing may be a more central risk factor for STBs than other mental health symptom dimensions in this age range, and intervention programs that provide age-appropriate treatment for internalizing symptoms could be valuable for reducing and preventing early onset STBs (Wasserman et al., 2021). Given its high centrality in the network model, intervening on internalizing symptoms might also have subsequent protective effects on other risk factors for STBs. Furthermore, results suggest that psychosocial interventions designed for early adolescents may benefit from prioritizing increased school support and engagement, as well as improving family support, engagement, and communication. Interventions that prevent or delay the initiation of recreational substance use may also have protective effects against STBs (Stockings et al., 2016). Universal school-based interventions have shown promise for protecting against mental health concerns broadly (e.g., Catalano, 2021), and early research suggests universally implemented programs can effectively integrate curricula focused on STBs specifically (Calear et al., 2016; Schilling et al., 2016; Wasserman et al., 2015). Continued development and evaluation of such school-based programs is recommended.

### Limitations and future directions

Results from the present study should be considered in the context of some limitations. First, while the ABCD data has many strengths, measurement waves are spaced approximately 1 year apart. Because the causal timescales between STBs and risk and protective factors are likely shorter than 1 year, measuring these constructs at shorter timescales would better capture proximal associations between variables (Harmer et al., 2021). Relatedly, although the ABCD study battery allowed for examination of a wide range of STB risk and protective factors, measures provided sparse detail about the specific timing, severity, and frequency of many constructs of interest.

Second, due to statistical assumptions of panel GVAR models, all study variables needed available data from three annual waves. Some relevant constructs were not included due to not having available data at all timepoints (e.g., impulsivity, peer experiences). Static risk factors that can influence long-term risk for suicide but were not repeatedly measured were also not considered (e.g., family history of suicide). In the case of our stressful life events variable, data for this construct was available from all three waves but the measures differed across timepoints (KSADS PTSD module at baseline and Year 2, LES at Years 1–2). Given the salience of stressful life events as a risk factor for youth STBs in extensive prior literature (Carballo et al., 2020; Cha et al., 2018; de Sousa et al., 2017), we elected to merge these measures to retain this construct in our models. Sensitivity analyses suggested this analytic decision did not introduce bias to the model. Nonetheless, results involving the stressful life events variable should be interpreted with this measurement limitation in mind, and replication of our results in other samples will increase confidence in how stressful life events relate to STBs and other risk and protective factors.

Third, analyses relied on either self- or parent-report data. We note that most of the direct associations observed for STBs (self-reported by youth) were with other variables youth self-reported on (family conflict, parental monitoring, school protective factors, and substance use). Similarly, many of the parent-report variables had strongest associations with other parent-report variables in the networks (mental health symptoms and life stressors). All the examined risk and protective factors have been robustly associated with youth STBs in previous research regardless of measure reporter (Carballo et al., 2020; Cha et al., 2018; de Sousa et al., 2017). Nonetheless, it is possible that bias for larger effects among variables from the same reporter could have influenced the observed network structures. Replicating this study using data from both parent and youth reports for each variable will increase confidence in results.

Fourth, despite the use of a school-based population-level sampling design to reduce selection bias, the ABCD sample includes overrepresentation of dominant social identities. Minoritized identity status and the intersectionality of multiple minoritized identities are associated with STBs (e.g., VanBronkhorst et al., 2021). Relations between STB risk and protective factors may also vary across social identity groups (Cha et al., 2018; Klonsky et al., 2017; Wiglesworth et al., 2022). Some groups of youth with elevated risk for STBs compared to the general population, such as youth involved in foster care, Child Protective Services, and the juvenile justice system (Teplin et al., 2015), are less likely to participate in population-based research studies (Feldstein Ewing et al., 2018; Sharma et al., 2021). Thus, replication of study results in more diverse and underserved populations of youth is warranted (Cha et al., 2018). Future work should also examine how minoritized identity status and experiences of minority stress and social safety influence the development and longitudinal course of STBs in this sample (e.g., Diamond & Alley, 2022).

## Conclusions

Despite the aforementioned limitations, the present study provides valuable information about potential risk and protective pathways for STBs among early adolescents. There is a critical need for research focusing on STBs during this developmental stage, as rates of youth STBs are rising and early adolescents have received relatively little attention in STB literature compared to older age groups (Ayer et al., 2020; Nock et al., 2013; de Sousa et al., 2017). This study represents a novel extension of the network approach to psychopathology to increase understanding of early onset STBs. Results emphasize that family and school experiences are salient social risk factors for STBs in early adolescents. Additionally, internalizing problems appear to be a more important risk factor than other mental health symptoms in this age range, and internalizing could possibly be a pathway through which stressful life events and other mental health symptoms contribute to STBs. Substance use was also associated with elevated risk for STBs. Results also suggest the potential for feedback systems of risk, in which the activation of multiple risk factors might exacerbate risk for STBs. Age-specific early interventions for STBs may benefit from focusing on increased social support in family and school domains, identifying and intervening on internalizing symptoms, and preventing early onset substance use.

## Supplementary Material

Supplementary material

## Figures and Tables

**Table 1.** Sample demographic characteristics and STB endorsement across study timepoints

	Analytic sample (*N* = 9,854)^1^ Mean (*SD*) or *n* (%)			Original ABCD sample (*N* = 11,876)^2^ Mean (*SD*) or *n* (%)		
	Baseline	Year 1	Year 2	Baseline	Year 1	Year 2
Age (in years)	9.90 (0.62)	10.91 (0.63)	11.99 (0.66)	9.91 (0.62)	10.92 (0.64)	12.00 (0.66)
*n* unknown	0	582	1,250	0	0	0
Race						
AIAN/NHPI	66 (0.7%)	60 (0.7%)	55 (0.6%)	78 (0.7%)	72 (0.7%)	66 (0.6%)
Asian	249 (2.6%)	236 (2.6%)	215 (2.5%)	275 (2.3%)	261 (2.4%)	236 (2.3%)
Black	1,576 (16%)	1,398 (15%)	1,242 (15%)	1,869 (16%)	1,672 (15%)	1,494 (15%)
Mixed	1,194 (12%)	1,116 (12%)	1,046 (12%)	1,434 (12%)	1,347 (12%)	1,256 (12%)
Other	467 (4.8%)	424 (4.6%)	393 (4.6%)	525 (4.5%)	476 (4.3%)	444 (4.3%)
White	6,160 (63%)	5,913 (65%)	5,535 (65%)	7,524 (64%)	7,244 (65%)	6,773 (66%)
*n* unknown	142	707	1,368	171	153	145
Annual household income^3^						
<50K	2,746 (31%)	–	–	3,223 (30%)	–	–
≥50 & <100K	2,535 (28%)	–	–	3,071 (28%)	–	–
≥100K	3,707 (41%)	–	–	4,564 (42%)	–	–
*n* unknown	866	–	–	1,018	–	–
Highest education of parent^3^						
<HS diploma	510 (5.2%)	–	–	593 (5.0%)	–	–
HS diploma or GED	975 (9.9%)	–	–	1,132 (9.5%)	–	–
Some college	2,569 (26%)	–	–	3,079 (26%)	–	–
Bachelor	2,443 (25%)	–	–	3,015 (25%)	–	–
Postgrad degree	3,346 (34%)	–	–	4,043 (34%)	–	–
*n* unknown	11	–	–	14	–	–
Sex assigned at birth						
Female	4,671 (47%)	4,376 (47%)	4,054 (47%)	5,680 (48%)	5,353 (48%)	4,962 (48%)
Male	5,183 (53%)	4,896 (53%)	4,550 (53%)	6,196 (52%)	5,872 (52%)	5,452 (52%)
*n* unknown	0	582	1,250	0	0	0
“Are you gay or bisexual?”						
Yes	29 (0.3%)	127 (1.4%)	382 (4.5%)	39 (0.3%)	136 (1.2%)	427 (4.2%)
Maybe	94 (1.0%)	223 (2.4%)	337 (4.0%)	112 (0.9%)	257 (2.3%)	385 (3.8%)
No	7,276 (74%)	8,044 (87%)	7,475 (88%)	8,701 (73%)	9,765 (87%)	9,107 (89%)
Do not understand	2,440 (25%)	842 (9.1%)	275 (3.2%)	3,005 (25%)	1,022 (9.1%)	332 (3.2%)
*n* unknown	15	618	1,385	19	45	163
“Are you transgender?”						
Yes	11 (0.1%)	15 (0.2%)	37 (0.4%)	12 (0.1%)	16 (0.1%)	42 (0.4%)
Maybe	43 (0.4%)	80 (0.9%)	57 (0.7%)	46 (0.4%)	82 (0.7%)	64 (0.6%)
No	5,959 (61%)	7,477 (81%)	7,963 (93%)	7,112 (60%)	9,040 (81%)	9,637 (93%)
Do not understand	3,830 (39%)	1,680 (18%)	483 (5.7%)	4,691 (40%)	2,060 (18%)	590 (5.7%)
*n* unknown	11	602	1,314	15	27	81
Endorsed STBs since last measurement occasion						
Never	8,933 (91%)	8,404 (92%)	7,842 (92%)	10,763 (91%)	10,190 (92%)	9,517 (92%)
Ever	856 (8.7%)	759 (8.3%)	680 (8.0%)	1,040 (8.8%)	910 (8.2%)	796 (7.7%)
*n* unknown	65	691	1,332	73	125	101

Note:

1 The analytic sample.

2 The original ABCD sample, before randomly dropping sibling participants.

3 Variables only measured at baseline. Gender and sexual identities were self-reported by youth; all other demographics items were assessed by parent/caregiver report. Rates of missingness in the analytic sample increased over time due to study attrition. STBs = suicidal thoughts and behaviors; AIAN/NHPI = American Indian/Alaska Native or Native Hawaiian and other Pacific Islander; HS = high school.

**Table 2.** ABCD measures, reporter, and data availability of variables used in the current study

Variable	Measure	Reporter	Data availability
1. Suicidality	KSADS-COMP Suicide Module	Youth	Baseline, Year 1, Year 2
2. Internalizing	Child Behavior Checklist	Parent	Baseline, Year 1, Year 2
3. Social problems	Child Behavior Checklist	Parent	Baseline, Year 1, Year 2
4. Thought problems	Child Behavior Checklist	Parent	Baseline, Year 1, Year 2
5. Attention problems	Child Behavior Checklist	Parent	Baseline, Year 1, Year 2
6. Externalizing	Child Behavior Checklist	Parent	Baseline, Year 1, Year 2
7. Sleep problems	Sleep Disturbances Scale for Children	Parent	Baseline, Year 1, Year 2
8. Family conflict	PhenX Family Environment Scale-Family Conflict Subscale	Youth	Baseline, Year 1, Year 2
9. Parental monitoring	Parental Monitoring Survey	Youth	Baseline, Year 1, Year 2
10. Neighborhood safety	PhenX Neighborhood Safety/Crime Survey	Parent	Baseline, Year 1, Year 2
11. School protective factors	PhenX School Risk & Protective Factors Survey	Youth	Baseline, Year 1, Year 2
12. Stressful life events^1^	KSADS-COMP PTSD Module	Parent	Baseline, Year 2
	Life Events Scale	Parent	Year 1, Year 2
13. Material hardship	PhenX Demographics Survey	Parent	Baseline, Year 1, Year 2
14. Substance use	Substance Use Timeline Follow-Back Survey	Youth	Baseline, Year 1, Year 2

Note:

1 Due to inconsistent data availability over time, stressful life events were coded as a composite score of items measuring traumatic and/or negative significant life events from the KSADS PTSD module and Life Events Scale (see Measures).

**Table 3.** Unstandardized descriptive statistics and missingness for variables across study timepoints (*N* = 9,854)

Variable	Baseline Mean (*SD*)	Year 1 Mean (*SD*)	Year 2 Mean (*SD*)
Suicidality	0.17 (0.74)	0.18 (0.80)	0.18 (0.79)
*n* missing	71	694	1,337
Internalizing	1.67 (1.81)	1.71 (1.83)	1.64 (1.84)
*n* missing	8	601	1,300
Social problems	1.67 (2.32)	1.55 (2.22)	1.34 (2.10)
*n* missing	8	601	1,300
Thought problems	1.18 (1.74)	1.15 (1.74)	0.99 (1.64)
*n* missing	8	601	1,300
Attention problems	3.08 (3.55)	2.98 (3.49)	2.81 (3.36)
*n* missing	8	601	1,300
Externalizing	2.27 (2.96)	2.14 (2.86)	1.98 (2.76)
*n* missing	8	601	1,300
Sleep problems	36.83 (8.36)	36.96 (8.22)	36.59 (8.15)
*n* missing	26	617	1,315
Family conflict	2.02 (1.95)	1.86 (1.84)	1.88 (1.80)
*n* missing	29	598	1,284
Parental monitoring	3.07 (2.58)	2.57 (2.27)	2.57 (2.33)
*n* missing	24	591	1,281
Neighborhood safety	3.40 (2.94)	3.39 (2.87)	3.43 (2.81)
*n* missing	45	625	1,352
School protective factors	39.22 (5.45)	39.86 (5.24)	38.30 (5.42)
*n* missing	29	592	1,283
Stressful life events			
KSADS PTSD Module	0.51 (0.93)	–	0.47 (0.78)
Life Events Scale	–	1.04 (1.57)	1.05 (1.61)
*n* missing	242	585	1,717
Material hardship	0.48 (1.10)	0.46 (1.10)	0.38 (0.95)
*n* missing	115	677	1,379
Substance use	0.23 (0.43)	0.10 (0.31)	0.11 (0.34)
*n* missing	23	621	1,295

*Note:* Rates of missingness increased over time due to study attrition. *SD* = standard deviation.

**Table 4.** Multilevel correlations and intraclass correlations for study variables in the full sample (*N* = 9,854)

Variable	1	2	3	4	5	6	7	8	9	10	11	12	13	14
Between-subject correlations														
1. Suicidality	1.00													
2. Internalizing	0.19	1.00												
3. Social problems	0.19	0.65	1.00											
4. Thought problems	0.16	0.58	0.59	1.00										
5. Attention problems	0.17	0.52	0.63	0.62	1.00									
6. Externalizing	0.19	0.58	0.66	0.57	0.66	1.00								
7. Sleep problems	0.13	0.54	0.47	0.48	0.48	0.48	1.00							
8. Family conflict	0.20	0.11	0.19	0.14	0.19	0.24	0.11	1.00						
9. Parental monitoring	0.19	0.10	0.16	0.14	0.23	0.16	0.09	0.35	1.00					
10. Neighborhood safety	0.03	0.12	0.17	0.09	0.12	0.15	0.14	0.11	0.09	1.00				
11. School protective factors	−0.21	−0.13	−0.14	−0.13	−0.22	−0.18	−0.14	−0.30	−0.45	−0.03	1.00			
12. Stressful life events	0.11	0.31	0.29	0.25	0.25	0.30	0.27	0.12	0.07	0.13	−0.07	1.00		
13. Material hardship	0.09	0.18	0.25	0.15	0.19	0.22	0.20	0.18	0.14	0.31	−0.04	0.29	1.00	
14. Substance use	0.12	0.02	0.00	0.05	0.06	0.06	0.05	0.04	0.05	−0.07	−0.14	0.02	−0.06	1.00
**Within-subject correlations**														
1. Suicidality	1.00													
2. Internalizing	0.04	1.00												
3. Social problems	0.02	0.40	1.00											
4. Thought problems	0.01	0.34	0.30	1.00										
5. Attention problems	0.02	0.35	0.35	0.32	1.00									
6. Externalizing	0.04	0.42	0.41	0.34	0.44	1.00								
7. Sleep problems	0.01	0.23	0.17	0.17	0.19	0.20	1.00							
8. Family conflict	0.07	0.03	0.02	0.02	0.04	0.04	0.02	1.00						
9. Parental monitoring	0.05	0.03	0.03	0.02	0.04	0.05	0.02	0.13	1.00					
10. Neighborhood safety	0.00	0.03	0.02	0.02	0.03	0.03	0.05	0.00	−0.01	1.00				
11. School protective factors	−0.05	−0.05	−0.03	−0.02	−0.05	−0.06	−0.03	−0.13	−0.19	0.00	1.00			
12. Stressful life events	0.01	0.10	0.08	0.05	0.06	0.07	0.06	0.01	0.00	0.01	−0.02	1.00		
13. Material hardship	−0.01	0.08	0.06	0.05	0.06	0.07	0.04	0.00	−0.01	0.01	0.01	0.03	1.00	
14. Substance use	0.05	0.00	0.02	0.02	0.01	0.02	−0.02	0.04	0.07	−0.01	−0.04	0.01	0.01	1.00
**Intraclass correlation**														
	0.28	0.67	0.66	0.63	0.75	0.72	0.66	0.44	0.41	0.62	0.46	0.37	0.57	0.30

*Note*: Correlations represent Spearman’s rho coefficients, estimated using the *psych* package (Revelle, 2024).

**Table 5.** Estimated directed partial correlations for the temporal network (*N* = 9,854)

Variable	1	2	3	4	5	6	7	8	9	10	11	12	13	14
1. Suicidality	0.087	–	–	–	–	–	–	–	–	–	–	–	–	–
2. Internalizing	–	0.085	–	–	−0.023	−0.023	–	–	–	–	–	–	–	–
3. Social problems	–	–	0.077	–	–	0.012	–	–	–	–	–	–	–	–
4. Thought problems	–	–	–	0.082	–	0.025	–	–	–	–	–	–	−0.035	–
5. Attention problems	–	–	–	–	0.132	–	–	–	–	–	–	–	0.047	–
6. Externalizing	–	0.036	0.068	0.059	0.054	0.156	–	–	–	–	–	–	–	–
7. Sleep problems	–	0.024	0.021	0.030	0.030	–	0.193	–	–	–	–	–	–	–
8. Family conflict	–	0.014	–	–	–	0.022	–	0.142	0.049	–	−0.039	–	–	–
9. Parental monitoring	–	–	–	–	–	–	–	0.037	0.155	–	–	–	–	–
10. Neighborhood safety	–	–	–	–	–	–	–	–	–	0.116	–	–	–	–
11. School protective factors	–	−0.012	−0.033	–	−0.022	–	–	−0.029	–	–	0.160	–	–	–
12. Stressful life events	–	–	–	–	–	–	–	–	–	–	–	−0.057	–	–
13. Material hardship	–	–	–	−0.027	0.036	–	–	–	–	–	–	0.057	0.130	–
14. Substance use	–	–	–	–	–	–	–	–	–	–	–	–	–	0.112

*Note*: Values represent lagged and autoregressive (i.e., across timepoints) directed partial correlations at the within-person level. Values on the diagonal are autocorrelations. The *psychonetrics* package does not currently provide standard errors for partial directed correlations (Epskamp, 2022). See Figure 1a for graphical representation of the temporal network structure.

**Table 6.** Estimated undirected partial correlations for the contemporaneous (lower triangle) and between-subjects (upper triangle) networks (*N* = 9,854)

Variable	1	2	3	4	5	6	7	8	9	10	11	12	13	14
1. Suicidality	NA	0.133 (0.016)	–	–	–	–	–	0.144 (0.023)	0.117 (0.030)	–	−0.102 (0.029)	0.094 (0.020)	–	0.160 (0.027)
2. Internalizing	0.052 (0.006)	NA	0.413 (0.012)	0.212 (0.015)	−0.123 (0.015)	–	0.334 (0.014)	−0.094 (0.016)	−0.078 (0.015)	–	−0.107 (0.016)	0.102 (0.014)	−0.051 (0.015)	−0.064 (0.013)
3. Social problems	–	0.266 (0.007)	NA	0.190 (0.016)	0.257 (0.014)	0.287 (0.013)	−0.080 (0.017)	0.055 (0.017)	–	0.063 (0.012)	0.064 (0.013)	–	0.102 (0.014)	–
4. Thought problems	–	0.188 (0.007)	0.106 (0.008)	NA	0.381 (0.013)	0.092 (0.016)	0.109 (0.018)	–	–	−0.052 (0.012)	–	–	–	–
5. Attention problems	–	0.091 (0.008)	0.145 (0.007)	0.158 (0.007)	NA	0.261 (0.014)	0.142 (0.016)	−0.077 (0.017)	0.214 (0.013)	–	–	–	−0.096 (0.013)	–
6. Externalizing	–	0.186 (0.009)	0.261 (0.009)	0.199 (0.009)	0.289 (0.008)	NA	0.114 (0.014)	0.190 (0.016)	−0.077 (0.014)	–	–	0.134 (0.012)	–	0.089 (0.013)
7. Sleep problems	–	0.137 (0.008)	0.026 (0.008)	0.052 (0.008)	0.074 (0.008)	0.037 (0.008)	NA	–	–	0.026 (0.014)	–	0.083 (0.017)	0.117 (0.015)	–
8. Family conflict	0.060 (0.007)	–	–	–	–	0.026 (0.006)	–	NA	0.221 (0.034)	0.065 (0.014)	−0.141 (0.032)	–	0.091 (0.017)	–
9. Parental monitoring	0.040 (0.007)	–	–	–	–	0.017 (0.006)	–	0.122 (0.010)	NA	–	−0.531 (0.020)	−0.117 (0.020)	0.196 (0.021)	−0.111 (0.025)
10. Neighborhood safety	–	–	0.024 (0.006)	–	–	–	0.041 (0.007)	–	–	NA	–	0.037 (0.016	0.296 (0.014)	−0.109 (0.017)
11. School protective factors	−0.038 (0.007)	–	−0.019 (0.007)	0.029 (0.007)	−0.041 (0.007)	−0.026 (0.007)	–	−0.134 (0.010)	−0.193 (0.007)	–	NA	−0.069 (0.020)	0.128 (0.020)	−0.185 (0.024)
12. Stressful life events	–	0.069 (0.007)	0.031 (0.007)	–	–	–	0.036 (0.008)	–	–	–	–	NA	0.318 (0.017)	–
13. Material hardship	–	0.051 (0.007)	–	–	0.051 (0.008)	–	–	–	−0.031 (0.007)	–	–	0.055 (0.008)	NA	−0.049 (0.018)
14. Substance use	0.039 (0.007)	–	–	–	–	–	–	0.023 (0.007)	0.031 (0.007)	–	−0.054 (0.007)	–	–	NA

*Note*: Lower triangle values represent contemporaneous (i.e., same measurement occasion) undirected partial correlations at the within-person level after adjusting for temporal effects and other variables in the model. Upper triangle values represent undirected partial correlations at the between-person level (i.e., associations between overall variable means) after adjusting for other variables in the model. Standard errors are provided in parentheses. See Figures 1b–c for graphical representation of the contemporaneous and between-subjects network structures.

## Data Availability

The data the support the findings of this study are available from the National Institute of Mental Health Data Archive.
