# Supplementary material for: Longitudinal panel networks of risk and protective factors for early adolescent suicidality in the ABCD sample
Source: Dev Psychopathol. Author manuscript; Available in PMC 2025 Sep 5. (PMC12108376; doi:10.1017/S0954579424001597)
Supplement: Supplementary material [file NIHMS2081652-supplement-Supplementary_material.docx]

**Appendix**

Child Behavior Checklist Scoring

Anxious/depressive = cbcl_q14_p + cbcl_q29_p + cbcl_q30_p + cbcl_q31_p + cbcl_q32_p + cbcl_q33_p + cbcl_q35_p + cbcl_q45_p + cbcl_q50_p + cbcl_q52_p + cbcl_q71_p + cbcl_q112_p

Withdrawn/depressed = cbcl_q05_p + cbcl_q42_p + cbcl_q65_p + cbcl_q69_p + cbcl_q75_p + cbcl_q102_p + cbcl_q103_p + cbcl_q111_p

Somatic = cbcl_q49_p + cbcl_q51_p + cbcl_q54_p + cbcl_q56a_p + cbcl_q56b_p + cbcl_q56c_p + cbcl_q56d_p + cbcl_q56e_p + cbcl_q56f_p + cbcl_q56g_p

Social = cbcl_q11_p + cbcl_q12_p + cbcl_q25_p + cbcl_q27_p + cbcl_q34_p + cbcl_q36_p + cbcl_q38_p + cbcl_q48_p + cbcl_q62_p + cbcl_q64_p + cbcl_q79_p

Thought = cbcl_q09_p + cbcl_q40_p + cbcl_q46_p + cbcl_q58_p + cbcl_q59_p + cbcl_q60_p + cbcl_q66_p + cbcl_q70_p + cbcl_q83_p + cbcl_q84_p + cbcl_q85_p

Attention = cbcl_q01_p + cbcl_q04_p + cbcl_q08_p + cbcl_q10_p + cbcl_q13_p + cbcl_q17_p + cbcl_q41_p + cbcl_q61_p + cbcl_q78_p + cbcl_q80_p

Rule Breaking = cbcl_q26_p + cbcl_q28_p + cbcl_q39_p + cbcl_q43_p + cbcl_q63_p + cbcl_q67_p + cbcl_q72_p + cbcl_q73_p + cbcl_q81_p + cbcl_q82_p + cbcl_q90_p + cbcl_q96_p + cbcl_q101_p + cbcl_q106_p

Aggressive = cbcl_q03_p + cbcl_q16_p + cbcl_q19_p + cbcl_q20_p + cbcl_q21_p + cbcl_q22_p + cbcl_q23_p + cbcl_q37_p + cbcl_q57_p + cbcl_q68_p + cbcl_q86_p + cbcl_q87_p + cbcl_q88_p + cbcl_q89_p + cbcl_q94_p + cbcl_q95_p + cbcl_q97_p + cbcl_q104_p

| Supplemental Table S1. Linear regression coefficients of each study variable regressed on youth age (*N* = 9,854) | |
| --- | --- |
| Variable | *B* (*SE*) |
| 1. Suicidality | 0.008 (0.006) |
| 2. Internalizing | 0.005 (0.006) |
| 3. Social problems | -0.062 (0.006) ** |
| 4. Thought problems | -0.044 (0.006) ** |
| 5. Attention problems | -0.031 (0.006) ** |
| 6. Externalizing | -0.037 (0.006) ** |
| 7. Sleep problems | -0.007 (0.006) |
| 8. Family conflict | -0.033 (0.006) ** |
| 9. Parental monitoring | -0.104 (0.006) ** |
| 10. Neighborhood safety | -0.007 (0.006) |
| 11. School protective factors | -0.074 (0.006) ** |
| 12. Stressful life events | -0.005 (0.006) |
| 13. Material hardship | -0.036 (0.006) ** |
| 14. Substance use | -0.077 (0.006) ** |
| *Note*: Resulting residuals for each variable were saved and modeled as nodes in the panel GVAR Model. Linear modeling was used despite non-normal variables to be consistent with parametric estimation in the panel GVAR models; model selection and bootstrapping procedures increase confidence that non-normality did not bias results (Epskamp, 2020). * *p* < 0.01, ***p* < 0.001. | |

| Supplemental Table S2. Number of times (out of 100) each edge was included in the Panel GVAR case-drop bootstrap resampling procedure | | | | | | | | | | | | | | |
| --- | --- | --- | --- | --- | --- | --- | --- | --- | --- | --- | --- | --- | --- | --- |
|  | 1 | 2 | 3 | 4 | 5 | 6 | 7 | 8 | 9 | 10 | 11 | 12 | 13 | 14 |
| Temporal network structure | | | | | | | | | | | | | | |
| 1. Suicidality | **92** | 8 | 0 | 7 | 17 | 5 | 0 | 4 | 1 | 0 | 1 | 2 | 0 | 0 |
| 2. Internalizing | 0 | **100** | 3 | 21 | **38** | **47** | 1 | 1 | 0 | 0 | 0 | 6 | 1 | 0 |
| 3. Social problems | 0 | 23 | **100** | 37 | 24 | **53** | 26 | 0 | 1 | 0 | 6 | 1 | 1 | 0 |
| 4. Thought problems | 3 | 15 | 16 | **100** | 23 | **62** | 11 | 0 | 0 | 0 | 1 | 0 | **66** | 0 |
| 5. Attention problems | 1 | 0 | 5 | 12 | **100** | 0 | 1 | 0 | 0 | 0 | 0 | 7 | **36** | 0 |
| 6. Externalizing | 0 | **9** | **64** | **19** | **15** | **100** | 13 | 2 | 2 | 0 | 2 | 35 | 0 | 0 |
| 7. Sleep problems | 3 | **85** | **77** | **82** | **64** | 22 | **100** | 1 | 0 | 0 | 16 | 9 | 28 | 4 |
| 8. Family conflict | 19 | **44** | 0 | 0 | 2 | **68** | 0 | **100** | **76** | 0 | **57** | 5 | 0 | 0 |
| 9. Parental monitoring | 10 | 0 | 0 | 0 | 0 | 1 | 0 | **27** | **100** | 0 | 0 | 0 | 0 | 1 |
| 10. Neighborhood safety | 15 | 0 | 0 | 6 | 3 | 22 | 5 | 1 | 0 | **100** | 3 | 0 | 1 | 0 |
| 11. School protective factors | 0 | **29** | **100** | 1 | **57** | 11 | 7 | **41** | 13 | 0 | **100** | 6 | 2 | 0 |
| 12. Stressful life events | 0 | 22 | 38 | 2 | 1 | 0 | 2 | 0 | 1 | 5 | 0 | **94** | 0 | 0 |
| 13. Material hardship | 0 | 0 | 0 | **70** | **19** | 0 | 10 | 0 | 0 | 3 | 0 | **99** | **100** | 0 |
| 14. Substance use | 2 | 1 | 0 | 0 | 0 | 0 | 0 | 0 | 7 | 0 | 11 | 0 | 0 | **100** |
| Contemporaneous (lower triangle) and between-subjects (upper triangle) network structures | | | | | | | | | | | | | | |
| 1. Suicidality | NA | **91** | 22 | 18 | 36 | 14 | 6 | **87** | **31** | 13 | **94** | **76** | 8 | **94** |
| 2. Internalizing | **100** | NA | **100** | **100** | **100** | 21 | **100** | **100** | **46** | 0 | **100** | **100** | **63** | **75** |
| 3. Social problems | 1 | **100** | NA | **100** | **100** | **100** | **76** | **61** | 4 | **83** | **92** | 1 | **94** | 22 |
| 4. Thought problems | 4 | **100** | **100** | NA | **100** | **99** | **100** | 0 | 6 | **66** | 23 | 13 | 34 | 2 |
| 5. Attention problems | 11 | **100** | **100** | **100** | NA | **100** | **100** | **95** | **100** | 6 | 29 | 22 | **67** | 4 |
| 6. Externalizing | 2 | **100** | **100** | **100** | **100** | NA | **98** | **100** | **99** | 7 | 0 | **100** | 1 | **96** |
| 7. Sleep problems | 0 | **100** | **69** | **100** | **100** | **95** | NA | 3 | 35 | **57** | 27 | **84** | **100** | 17 |
| 8. Family conflict | **100** | 0 | 0 | 2 | 0 | **99** | 1 | NA | **100** | **67** | **95** | 32 | **80** | 0 |
| 9. Parental monitoring | **99** | 0 | 9 | 0 | 0 | **54** | 1 | **100** | NA | 31 | **100** | **69** | **100** | **44** |
| 10. Neighborhood safety | 0 | 0 | **44** | 1 | 1 | 33 | **97** | 0 | 0 | NA | 2 | **54** | **100** | **100** |
| 11. School protective factors | **96** | 14 | **47** | **59** | **97** | **70** | 5 | **100** | **100** | 0 | NA | **24** | **95** | **100** |
| 12. Stressful life events | 3 | **100** | **73** | 0 | 9 | 8 | **96** | 0 | 1 | 0 | 0 | NA | **100** | 4 |
| 13. Material hardship | 0 | **100** | 0 | 17 | **86** | 50 | 23 | 0 | **85** | 6 | 2 | 100 | NA | **85** |
| 14. Substance use | **100** | 0 | 0 | 0 | 0 | 0 | 23 | **54** | **55** | 0 | **100** | 0 | 0 | NA |
| *Note*: Bold values represent edges that were included in the original analysis (Tables 5-6, Figure 1). Each of the 100 bootstrapped models used a random 75% of the full analytic sample (*n* = 7,388). | | | | | | | | | | | | | | |

Supplemental Figure 1. Node centrality metrics for the average network structures across the100 case-dropped bootstrapped models. Each of the 100 bootstrapped models used a random 75% of the full analytic sample (*n* = 7,388). Centrality metrics are shown in the metric of *z*-scores.
